# ctDNA response after pembrolizumab in non-small cell lung cancer: phase 2 adaptive trial results

**DOI:** 10.1038/s41591-023-02598-9

**Published:** 2023-10-09

**Authors:** Valsamo Anagnostou, Cheryl Ho, Garth Nicholas, Rosalyn Anne Juergens, Adrian Sacher, Andrea S. Fung, Paul Wheatley-Price, Scott A. Laurie, Benjamin Levy, Julie R. Brahmer, Archana Balan, Noushin Niknafs, Egor Avrutin, Liting Zhu, Mark Sausen, Penelope A. Bradbury, Jill O’Donnell-Tormey, Pierre Olivier Gaudreau, Keyue Ding, Janet Dancey

**Affiliations:** 1 Sidney Kimmel Comprehensive Cancer Center, Johns Hopkins University School of Medicine, Baltimore, MD USA; 2 Bloomberg-Kimmel Institute for Cancer Immunotherapy, Johns Hopkins University School of Medicine, Baltimore, MD USA; 3 BCCA-Vancouver Cancer Centre, Vancouver, BC Canada; 4 Ottawa Hospital Research Institute, Ottawa, ON Canada; 5 Juravinski Cancer Centre at Hamilton Health Sciences, Hamilton, ON Canada; 6 Princess Margaret Cancer Centre, University Health Network, Toronto, ON Canada; 7 Kingston Health Sciences Centre, Kingston, ON Canada; 8 Canadian Cancer Trials Group, Queen’s University, Kingston, ON Canada; 9 Personal Genome Diagnostics (LabCorp), Baltimore, MD USA; 10 Cancer Research Institute, New York, NY USA

**Keywords:** Predictive markers, Non-small-cell lung cancer, Medical genomics, Translational research

## Abstract

Circulating tumor DNA (ctDNA) has shown promise in capturing primary resistance to immunotherapy. BR.36 is a multi-center, randomized, ctDNA-directed, phase 2 trial of molecular response-adaptive immuno-chemotherapy for patients with lung cancer. In the first of two independent stages, 50 patients with advanced non-small cell lung cancer received pembrolizumab as standard of care. The primary objectives of stage 1 were to ascertain ctDNA response and determine optimal timing and concordance with radiologic Response Evaluation Criteria in Solid Tumors (RECIST) response. Secondary endpoints included the evaluation of time to ctDNA response and correlation with progression-free and overall survival. Maximal mutant allele fraction clearance at the third cycle of pembrolizumab signified molecular response (mR). The trial met its primary endpoint, with a sensitivity of ctDNA response for RECIST response of 82% (90% confidence interval (CI): 52–97%) and a specificity of 75% (90% CI: 56.5–88.5%). Median time to ctDNA response was 2.1 months (90% CI: 1.5–2.6), and patients with mR attained longer progression-free survival (5.03 months versus 2.6 months) and overall survival (not reached versus 7.23 months). These findings are incorporated into the ctDNA-driven interventional molecular response-adaptive second stage of the BR.36 trial in which patients at risk of progression are randomized to treatment intensification or continuation of therapy. ClinicalTrials.gov ID: NCT04093167.

## Abstract (web summary)

In the first stage of the BR.36 adaptive trial in patients with non-small cell lung cancer receiving anti-PD1 immunnotherapy, the primary endpoint of concordance between circulating tumor DNA (ctDNA) molecular response and RECIST response was met. The results will inform the second, ctDNA-directed stage.

## Main

Liquid biopsies are gaining momentum in immuno-oncology (IO) as they can be used to rapidly and accurately determine clinical response, especially in the metastatic setting^1,2^. As the landscape of IO-based therapies and clinical trials is expanding, we face emerging challenges related to heterogeneity in clinical responses and insufficiency of imaging to rapidly and accurately capture therapeutic response^3^. Furthermore, currently used predictive biomarkers, such as programmed death-ligand 1 (PD-L1) expression or tumor mutation burden (TMB), fail to consistently predict therapeutic response^4,5^. These challenges highlight the urgent unmet need to implement molecular response-driven approaches to interpret outcomes and guide therapy selection in IO. Liquid biopsy analyses of circulating cell-free tumor DNA (ctDNA) have shown promise in capturing tumor burden dynamics during immune checkpoint blockade, allowing patients with primary resistance to be rapidly identified and redirected to receive alternative therapies^1,6–14^. Minimally invasive dynamic ctDNA next-generation sequencing (NGS) analyses that can be used as an early endpoint of immunotherapy response may, thus, help guide therapy to maximize therapeutic benefit and minimize toxicity risks for patients^4^.

Focusing on patients with non-small cell lung cancer (NSCLC) as a representative example, where both pembrolizumab^15^ and combination pembrolizumab–carboplatin–taxane/pemetrexed^16^ are US Food and Drug Administration and Health Canada approved first-line treatment options, it is crucial to determine which patients would benefit from pembrolizumab monotherapy and which should receive combination immuno-chemotherapy. Such therapeutic decisions are not currently supported by either tumor PD-L1 or TMB status, introducing an unmet clinical need and an opportunity for dynamic assessments of ctDNA to guide treatment selection based on real-time tracking of circulating tumor burden. Nevertheless, several outstanding urgent questions need to be answered before implementation of liquid biopsy-guided ctDNA molecular responses in clinical decision-making. What is the best measure for tracking circulating tumor burden and which ctDNA features accurately capture survival outcomes? Should a tumor-informed or tumor-agnostic approach be implemented? What signifies a ctDNA molecular response and when does it occur? What is the concordance between ctDNA molecular response and Response Evaluation Criteria in Solid Tumors (RECIST) radiographic response? What are the patient subsets that would most benefit from a molecularly refined response assessment?

To address these challenging questions and further establish the role of ctDNA response as an early endpoint for clinical outcomes with immune checkpoint blockade, we designed a two-stage ctDNA-directed molecular response adaptive clinical trial, with the first stage focusing on answering the questions posed above and reported here.

## Results

### Study design

**Fig. 1:**
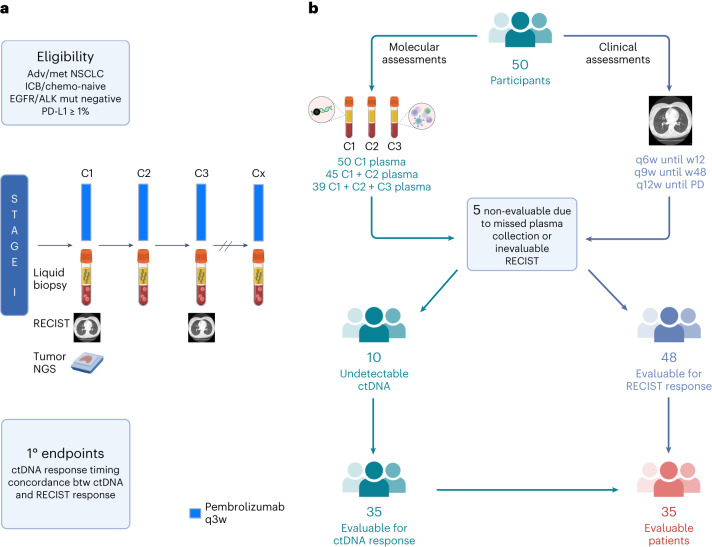
BR.36 trial schema. **a**, The first stage of the BR.36 trial enrolled patients with advanced/metastatic NSCLC who did not harbor clinically actionable genomic alterations in *EGFR* or *ALK* and had a PD-L1 expression level of ≥1%. Patients received pembrolizumab as per local standard of care, and RECIST radiographic response assessments were performed every 6 weeks until week 12 and at longer intervals thereafter (Methods). Serial liquid biopsies were collected before treatment administration on C1D1 (baseline), C2D1 (3 weeks) and C3D1 (6 weeks), followed by ctDNA molecular response assessments at these timepoints. The primary endpoints of the trial were to determine the optimal timepoint of ctDNA molecular response and validate the concordance of ctDNA molecular response with radiographic RECIST version 1.1 response. **b**, BR.36 reached its target enrollment of 50 patients; for each individual, serial radiographic assessments and liquid biopsy analyses were performed. C1D1 plasma was collected for all 50 patients; C1D1 and C2D1 plasma samples were collected for 45 patients; and plasma samples were collected for all three timepoints for 39 patients. Five patients were deemed not evaluable because of missed plasma collection or non-evaluable RECIST assessments. Of the 45 evaluable patients, 10 had undetectable ctDNA at all timepoints (no tumor-specific plasma variants detected), resulting in 35 patients with evaluable ctDNA and RECIST responses.

**Extended Data Fig. 1:**
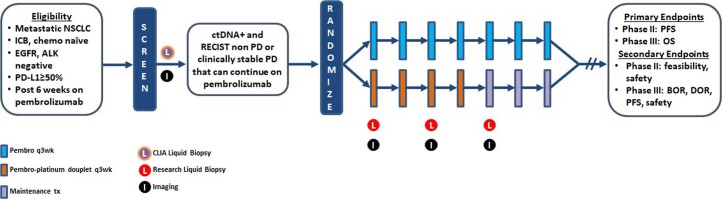
Trial schema for the interventional second stage of the BR.36 study. Abbreviations: NSCLC; non-small cell lung cancer, ICB; immune checkpoint blockade, chemo; chemotherapy, pembro; pembrolizumab, wk; week, PD; disease progression.

BR.36 is an international, multi-center, open-label, biomarker-directed phase 2 trial of ctDNA molecular response-adaptive immuno-chemotherapy for patients with treatment-naive NSCLC. The trial consists of two stages. In stage 1 (observational), patients with advanced/metastatic NSCLC who were eligible to receive standard-of-care single-agent pembrolizumab were enrolled in a single-arm study to evaluate, through serial liquid biopsy analyses, the optimal definition, timing and concordance of ctDNA molecular response with radiographic response (Fig. 1). The interventional randomized second stage of the trial will evaluate the potential clinical benefit of tailoring treatment to ctDNA molecular response (Extended Data Fig. 1). Key eligibility criteria included *EGFR* and *ALK* clinically actionable mutation-negative, immune checkpoint inhibitor-naive and chemotherapy-naive metastatic NSCLC with a PD-L1 expression level of ≥1% (Methods). Serial blood samples were obtained before treatment administration on cycle 1, day 1 (C1D1, pre-treatment), cycle 2, day 1 (C2D1, 3 weeks) and cycle 3, day 1 (C3D1, 6 weeks) for each study participant, and serial liquid biopsy analyses were performed for quantitative assessment of ctDNA dynamics. With the assumption that more than 80% of patients with metastatic NSCLC have detectable tumor-derived mutations in plasma^6^, a sample size of 50 patients was estimated to ensure acceptable sensitivity and specificity for ctDNA molecular response (Methods).

### Study enrollment and participants

**Fig. 2:**
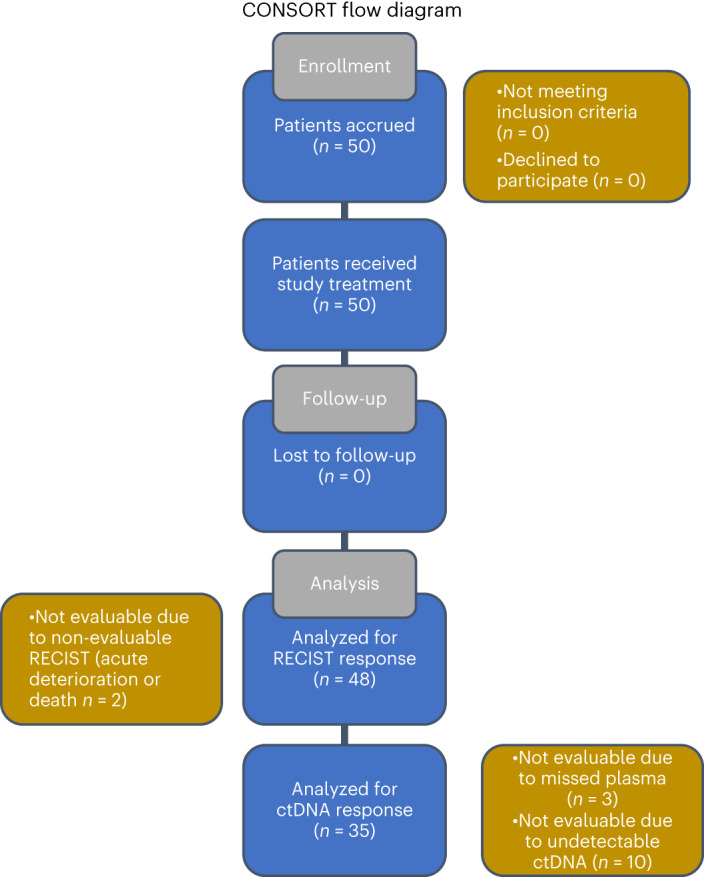
CONSORT flow diagram. Of the 50 patients enrolled, two were non-evaluable for RECIST response because of symptomatic progression/acute deterioration/death during cycle 1 without imaging (BR360020 and BR360021). The remaining 96% (48/50) of patients in the BR.36 study were evaluable for radiographic response assessment, which supports the feasibility of CT restaging in this population. Of the 48 patients with evaluable radiographic responses, three were non-evaluable because of missed plasma collection (BR360014, BR360016 and BR360029) due to withdrawn consent, rapid disease progression/death and protocol violation, respectively. Of the 45 patients evaluable for both radiographic and ctDNA responses, 22.2% (10/45) had undetectable ctDNA, which is consistent with previously reported ctDNA undetectable rate in patients with metastatic NSCLC (Anagnostou et al.^6^) and was within the CI of the undetectable ctDNA rate that we factored in the sample size calculations for the BR.36 stage 1 cohort. adv, advanced; btw, between; met, metastatic; mut, mutation; q6w, once every 6 weeks; q9w, once every 9 weeks; q12w, once every 12 weeks; w, week.

The trial was centrally activated on 17 October 2019 and was closed to accrual on 5 April 2022 after reaching target enrollment (study completion as per protocol). The first and last patients were enrolled on trial on 26 May 2020 and 5 April 2022, respectively. A total of 50 patients were accrued to the trial; all patients were followed for ctDNA molecular response and clinical response for a minimum of 12 weeks, and the clinical trial database was locked on 20 September 2022 (Fig. 1). Patient disposition and CONSORT diagram are described in detail in Fig. 2; there were two major protocol violations due to divergent timing of laboratory or imaging assessments (Methods). Median follow-up time was 13.5 months (range, 2.5–23.0 months; 95% confidence interval (CI): 6.9–14.5 months). Most patients were ever-smokers (98%), had stage IV NSCLC (98%) and had no prior systemic therapy (92%). The trial cohort consisted of 82% White, 52% female and 56% aged 65 years or older, and 76% of participants had an Eastern Cooperative Oncology Group performance status (ECOG PS) of 1 (Table 1). Seventy-six percent of tumors were adenocarcinomas, and 96% had a PD-L1 tumor proportion score (TPS) of ≥50%. Adverse events noted were within the expected spectrum of immune-related adverse events historically reported—one grade 4 event and 20 grade 3 events (Supplementary Tables 1 and 2). The most frequent serious adverse event was pneumonitis, reported in five (10%) patients. Four grade 5 events were reported, two due to disease progression and two possibly related to drug toxicity. Demographic, clinical, radiographic and pathological characteristics are summarized in Table 1 and Supplementary Table 3.

**Table 1:** Demographics, clinicopathologic characteristics and outcomes for the BR36 cohort (all enrolled patients)

Characteristic	Number (%)
Sex	
Female	26 (52.0)
Male	24 (48.0)
Race	
White	41 (82.0)
Black	2 (4.0)
Asian	4 (8.0)
Not Reported	3 (6.0)
Age^a^	
<65 years	22 (44.0)
≥65 years	28 (56.0)
ECOG PS	
0	12 (24.0)
1	38 (76.0)
Smoking status (current)	
Yes	12 (24.0)
No	37 (74.0)
Missing	1 (2.0)
Smoking history	
>100 cigarettes during lifetime	49 (98.0)
Missing	1 (2.0)
Prior systemic therapy	
No	46 (92.0)
Yes	4 (8.0)
Prior radiation	
No	27 (54.0)
Yes	23 (46.0)
Prior surgery	
No	1 (2.0)
Yes	49 (98.0)
Stage	
Stage III^b^	1 (2.0)
Stage IV	49 (98.0)
Histology	
Adenocarcinoma	38 (76.0)
Squamous cell carcinoma	6 (12.0)
Other	6 (12.0)
PD-L1 expression	
≥50% TPS	48 (96.0)
1–49%	2 (4.0)
RECIST response	
CR/PR	16 (32.0)
SD/PD	34 (68.0)
ctDNA response	
mR	15 (42.9)
mPD	20 (57.10)

^a^Median age was 65.5 years (range, 50–87 years).

^b^Not a candidate for surgical resection or definitive chemoradiation.

### Endpoints

The primary objectives of the study were to establish the concordance of ctDNA molecular response with radiographic RECIST/immune RECIST (iRECIST) response and define the optimal timepoint of ctDNA molecular response. Secondary objectives included the association of ctDNA molecular response with progression-free survival (PFS) and overall survival (OS), the correlation of depth of ctDNA kinetics with radiographic response and analyses of time to ctDNA molecular response.

### Radiographic and ctDNA molecular response

Best overall radiographic response was evaluated using RECIST and iRECIST at 12 weeks (Methods), and tumor responses were classified as RECIST/iRECIST response (complete response (CR/iCR) and partial response (PR/iPR)) and no RECIST response (stable disease (SD/iSD) and progressive disease (PD/iPD)) (Supplementary Table 4). The best overall RECIST response rate was 32% (90% CI: 21–44%), which was significantly less than the presumed 45% (*P* = 0.04; Methods), whereas the best overall iRECIST response rate was 36% (90% CI: 25–49%), which was not significantly different than the presumed 45% (*P* = 0.13). Median duration of radiographic response was 10.1 months (90% CI: 5.6–19.2 months) and 9.7 months (90% CI: 5.6–10.7 months) for RECIST and iRECIST response, respectively.

**Fig. 3:**
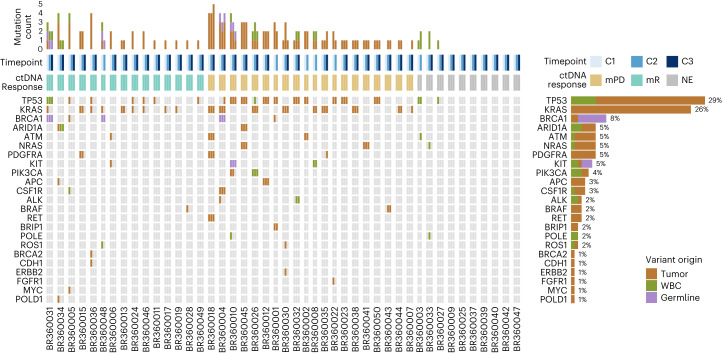
Overview of plasma variants detected by NGS. The total number, distribution and origin of variants detected by serial liquid biopsy analyses are shown for 45 patients with at least two serial liquid biopsies performed. A WBC DNA-informed approach allowed for classification of plasma variants by cellular origin and revealed that 17% of the plasma variants detected (14/82 variants) could be attributed to clonal hematopoiesis mutations. Frequently mutated genes included *TP53*, *KRAS*, *ARID1A*, *ATM*, *NRAS* and *PDGFRA*, which is consistent with the reported genomic landscape of NSCLC. Alteration prevalence for each gene is listed on the right. The mutation count per sample is displayed at the top, followed by rows indicating sample timepoint and ctDNA molecular response. Ten patients had undetectable tumor-derived mutations at all timepoints, rendering 35 patients evaluable for ctDNA molecular response. NE, not evaluable.

**Extended Data Fig. 2:**
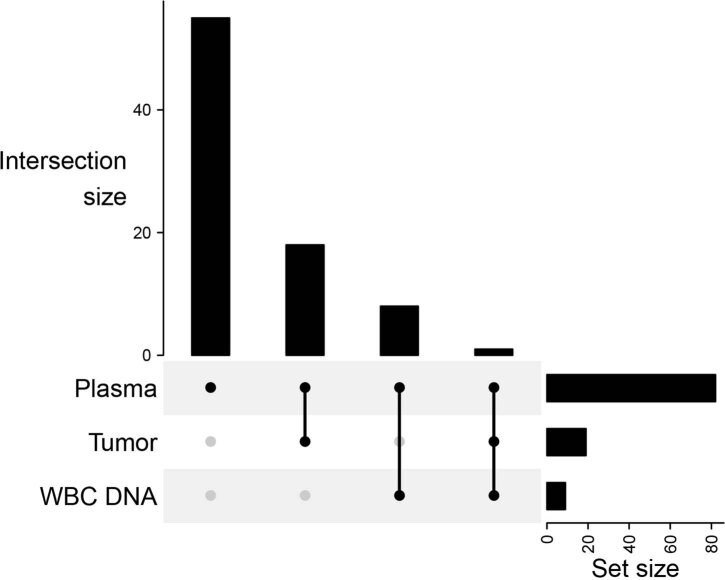
Intersection of variants detected by plasma, WBC DNA and tumor next-generation sequencing for the BR.36 study participants. UpSet plot for all plasma mutations detected (n = 82). From left to right the vertical bars represent the number of mutations that were detected only in the plasma (n = 55), detected in plasma and matched tumor (n = 18), detected in plasma and matched WBC DNA NGS (n = 8) and reported in plasma, tumor and matched WBC DNA NGS (n = 1). The horizontal bars on the right represent the total number of plasma mutations (n = 82), total plasma mutations in the tumor (n = 19) and total plasma mutations detected in the WBC DNA samples (n = 9).

With respect to ctDNA molecular response, we employed a tumor-agnostic, white blood cell (WBC) DNA-informed NGS approach and first determined the cellular origin of sequence variants detected in plasma (Methods and Supplementary Tables 5 and 6). Of the 82 unique plasma variants, 14 (17%) were confirmed to be clonal hematopoiesis derived; four (5%) were germline; and the remaining 64 (78%) were tumor derived (Fig. 3 and Extended Data Fig. 2). Germline and clonal hematopoiesis-related variants were subsequently excluded from downstream analyses. Expanding on the previously reported analytical performance of the NGS assay in contrived samples^17^, we employed a binomial model to calculate the estimated limit of detection based on the error-corrected coverage (Methods). These analyses revealed a median sensitivity of >99% and 93% for detection of an alteration occurring at 0.30% and 0.20% mutant allele fraction (MAF), respectively, which represented 95% (53/56) of tumor-specific variants detected at cycle 1 (Supplementary Table 6). Three well-characterized cancer hotspots were detected at a MAF of 0.14–0.2% at cycle 1, *KRAS* G12A with a MAF of 0.19% (BR360030), *NRAS* Q61L with a MAF of 0.14% (BR360041) and *KRAS* G12C with a MAF of 0.14% (BR360048), and, collectively, these findings support the sensitive detection of ctDNA mutations by the NGS assay used.

The maximal mutant allele fraction (maxMAF) of tumor-derived plasma mutations was tracked from the pre-treatment timepoint (C1D1) to on-therapy timepoints (C2D1 and C3D1), with maxMAF clearance signifying molecular response (mR), whereas persistence of maxMAF indicated molecular disease progression (mPD) (Supplementary Table 7)^6^. Among the 50 trial participants, five were not evaluable for ctDNA molecular response because of missed plasma collection or non-evaluable RECIST response; of the remaining 45 patients, 10 had undetectable ctDNA, and, as such, 35 patients (77.8%) were evaluable for ctDNA molecular response (Figs. 1 and 2). The undetectable ctDNA rate of 22.2% was not significantly different from the postulated 20% (*P* = 0.82). There were no differences in PFS or OS in the evaluable patient subset (*n* = 35) compared to the overall BR.36 study population (*n* = 50). Among the 35 evaluable patients, 15 were classified in the mR category, with an evaluable mR rate of 43% (90% CI: 0.29–0.58).

### Timing and concordance of ctDNA molecular response with radiographic response

**Fig. 4:**
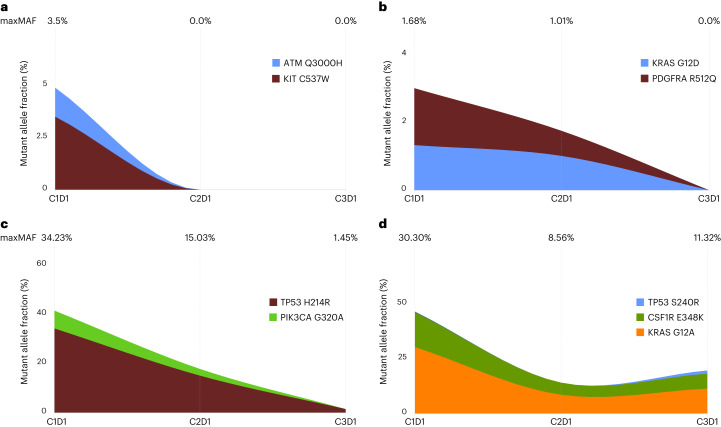
Representative ctDNA kinetics patterns. **a**–**d**, We identified four patterns of ctDNA kinetics: 13 patients showed ctDNA maxMAF clearance at C2D1, as shown here for patient BR360006 (**a**); two patients showed ctDNA maxMAF clearance at C3D1, as shown here for patient BR360015 (**b**); two patients had ctDNA reduction >85% but <100%, as shown here for patient BR360010 (**c**); and 18 patients showed ctDNA persistence throughout the timepoints analyzed, as shown here for patient BR360004 (**d**). Timepoints (C1D1, C2D1 and C3D1) are shown at the top of each panel, alongside the maxMAF of tumor-derived variants detected at each timepoint. Stacked area plots represent MAFs of individual tumor-specific variants as measured in liquid biopsies at baseline and on-therapy timepoints. Of note, the plot does not reflect the unknown hierarchical structure of tumor subclones harboring mutations, and, as such, the height of the area plot indicates the sum of MAFs from all mutations at each timepoint.

We identified four patterns of ctDNA kinetics: (1) ctDNA maxMAF clearance at C2D1, (2) maxMAF clearance at C3D1, (3) maxMAF reduction more than 85% in C2 or C3 and (4) ctDNA persistence (Fig. 4a–d). Of the 15 patients with ctDNA clearance, two showed ctDNA persistence at C2D1 and cleared ctDNA at C3D1 and, as such, were classified in the mR group (Fig. 4b). Two patients showed marked maxMAF reduction (>85% but <100%), and these patients were classified in the mPD group (Fig. 4c) The sensitivity of mR for RECIST best overall response (BOR) was 82% (90% CI: 52–97%), and the specificity was 75% (90% CI: 56.5–88.5%) (Table 2). The study met its primary endpoint for concordance between ctDNA and radiographic response, with both sensitivity and specificity better than the hypothesized 70%, and the lower 95% confidence bound of estimated sensitivity and specificity were higher than 50%. Median time to ctDNA molecular response was 2.1 months (90% CI: 1.5–2.6 months).

**Table 2:** Concordance between radiographic and ctDNA molecular response for patients evaluable for ctDNA molecular response

RECIST response (BOR)	Molecular response	
	mR	mPD
CR/PR	9 (82%)	2 (18%)
No RECIST response	6 (25%)	18 (75%)
**iRECIST response**		
iCR/iPR	10 (83%)	2 (17%)
No iRECIST response	5 (22%)	18 (78%)

**Fig. 5:**
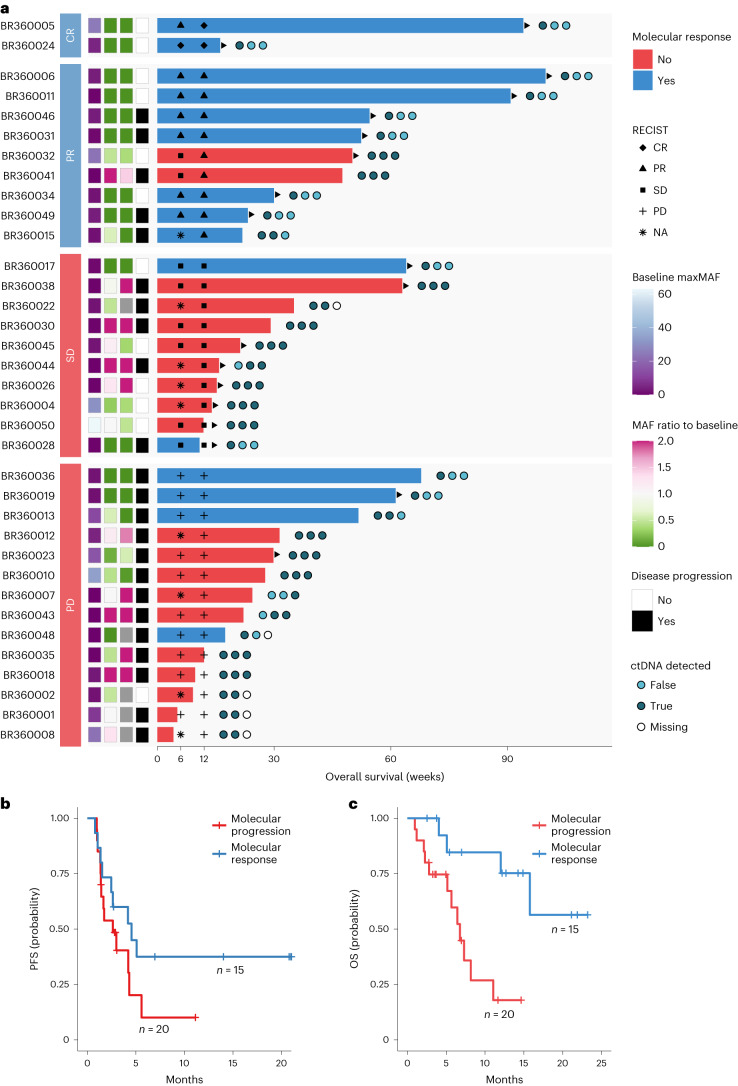
Analyses of PFS and OS by ctDNA molecular response. **a**, Swimmer plot depicting the timing of radiographic response assessment, molecular response trajectory and OS for each evaluable patient in the BR.36 stage 1 cohort. The patients are grouped by radiographic response category and ordered by OS within each group, where the bar color indicates the assigned molecular response. The circles to the right of each bar depict detection of ctDNA in the three liquid biopsy samples analyzed from timepoints C1D1, C2D1 and C3D1, from left to right, respectively. The three annotation columns to the left of the bars indicate the value of maxMAF in the baseline sample and the ratio of maxMAF in the C2 and C3 timepoints compared to the baseline. Gray tiles mark timepoints with no sample available for analysis. Triangles at the edge of survival intervals indicate ongoing follow-up. **b**,**c**, Patients with ctDNA mR had a longer PFS and OS compared to patients with mPD (5.03 months versus 2.6 months and not reached versus 7.23 months for PFS and OS, respectively; HR = 0.55, 95% CI: 0.27–1.13 and HR = 0.16, 95% CI: 0.05–0.50 for PFS and OS, respectively). NA, not applicable.

**Extended Data Fig. 3:**
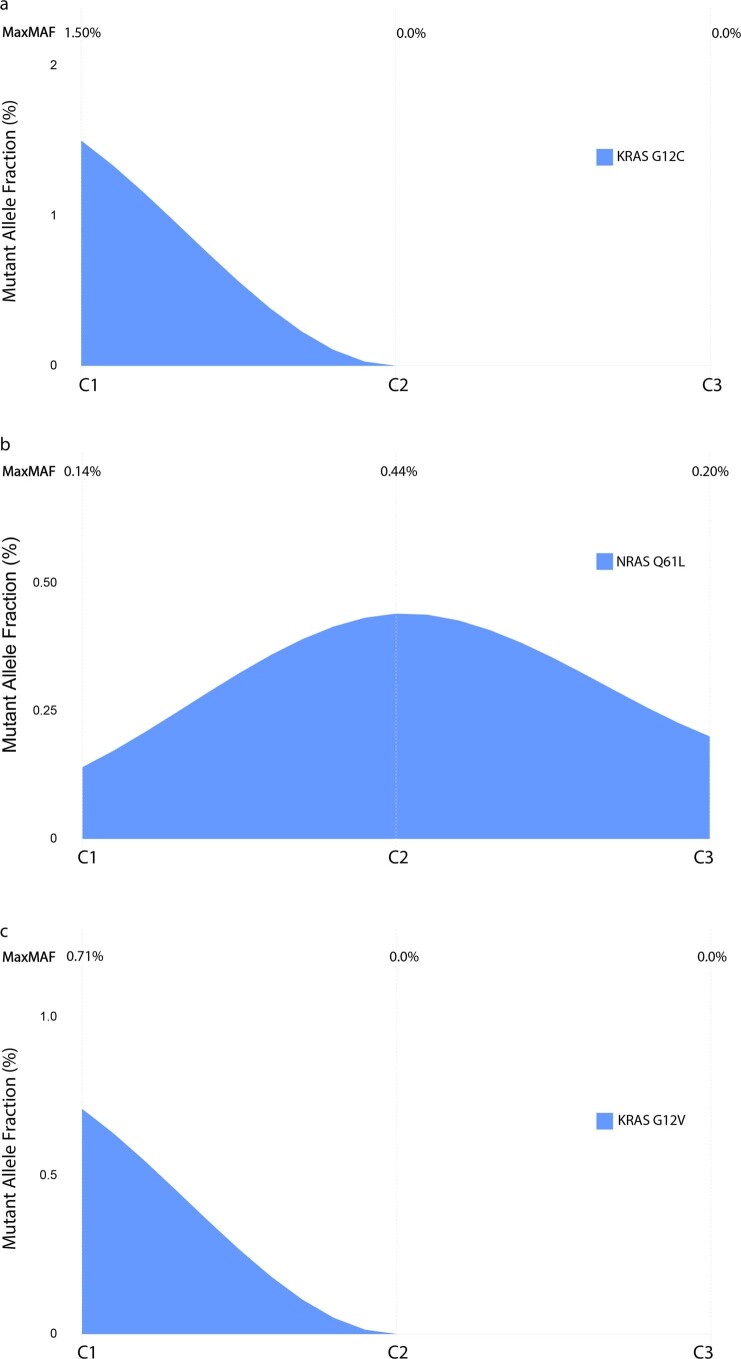
ctDNA trends for patients with discordant molecular and radiographic RECIST responses. (**a**) For patient BR360019, clearance of KRAS G12C at C2D1 signified ctDNA molecular response, which was discordant with the RECIST radiographic assessment of disease progression. (**b**) In contrast, patient BR360041 showed persistence of NRAS Q61L at C3D1 and was as such classified in the molecular disease progression category that while discrepant with a RECIST assessment of partial response, more accurately reflected a short progression-free survival of 1.47 months. (**c**) Notably, for patient BR360017 that had radiographically stable disease, clearance of KRAS G12V at C2D1 signified ctDNA molecular response and accurately captured the patient’s ongoing progression-free and overall survival of >13 months.

Although ctDNA and radiographic responses were overall concordant (Table 2 and Supplementary Table 7), there were several notable exceptions (Fig. 5a and Extended Data Fig. 3). Patient BR360019 had radiographic disease progression at 6 weeks and 12 weeks but showed ctDNA molecular response, which was reflective of the patient’s OS of 14.1 months (ongoing at the time of data lock). Patient BR360041 had radiographic partial response, which was discordant with ctDNA molecular disease progression, the latter better capturing a short radiographic response of 1.47 months (Fig. 5a and Extended Data Fig. 3). All patients with RECIST CR/PR at 6 weeks/C3D1 had ctDNA mR. Differential ctDNA molecular responses were noted for patients with stable RECIST assessments (*n* = 10); most patients with RECIST SD were classified in the mPD group (80%), with two patients showing mR. Of the two patients with mR/RECIST SD, BR360017 had adequate follow-up on the BR.36 trial to assess long-term clinical outcome, and, notably, ctDNA accurately reflected an ongoing PFS and OS of more than 13 months (Fig. 5a). In assessing radiographic response by iRECIST, the sensitivity of molecular response for iRECIST response was 83% (90% CI: 56–97%), and the specificity was 78% (90% CI: 60–91%) (Table 2); the lower 95% confidence bound of sensitivity and specificity were both better than 50%.

As part of post hoc analyses, we evaluated the concordance between ctDNA and radiographic responses in subsets of patients based on the level of maxMAF at cycle 1. Using 3% as a threshold for baseline maxMAF, sensitivity and specificity were 80% and 69% for maxMAF <3% and 83% and 88% for max MAF ≥3%, respectively. Using 4% as a threshold for baseline maxMAF, sensitivity and specificity were 88% and 69% for maxMAF <4% and 67% and 88% for maxMAF ≥4%, respectively. These analyses, although limited in power, further support the stability of ctDNA response assessment over a broad range of MAFs (Supplementary Table 8). Taken together, these findings suggest that, whereas ctDNA responses are largely concordant with radiographic RECIST responses, molecular assessments of circulating tumor burden dynamics may rapidly and more accurately capture long-term clinical outcomes, which can be particularly informative in the heterogeneous group of patients with radiographically stable disease.

### Correlation of ctDNA molecular response with secondary endpoints

**Extended Data Fig. 4:**
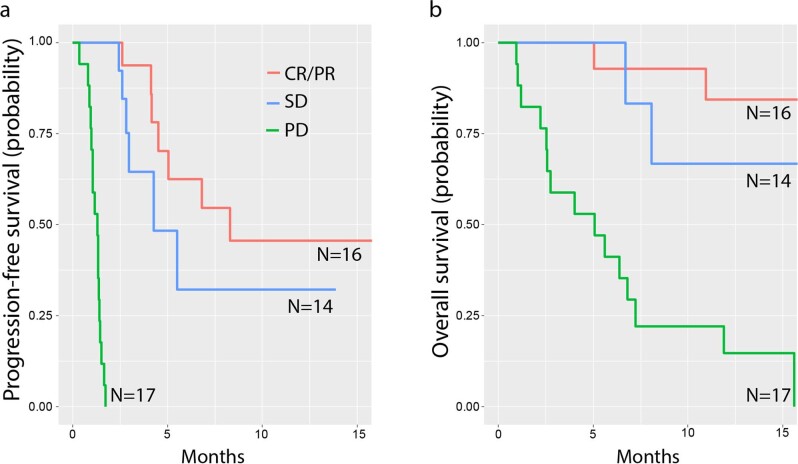
Progression-free and overall survival prognostication based on radiographic RECIST response for all patients on BR.36 with evaluable RECIST responses. RECIST response at 12 weeks less clearly distinguished patients with complete/partial responses compared to patients with stable disease for progression-free (8.31 vs 4.27 months, shown in panel **a**) and overall survival (not reached vs 16.89 months, shown in panel **b**).

**Extended Data Fig. 5:**
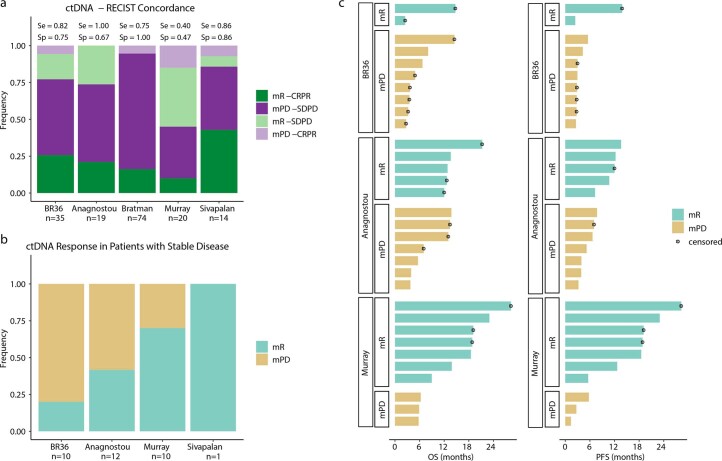
Heterogeneity of radiographic stable disease with respect to ctDNA response and long-term clinical outcomes. To expand the post hoc analyses of differential outcomes based on ctDNA response for patients with radiographically stable disease on immune checkpoint blockade, we computed the concordance between radiographic and ctDNA responses from previously reported IO cohorts together with BR.36 (Anagnostou et al., Cancer Research, 2019, Bratman et al., Nature Cancer, 2020, Murray et al., Cancer Research, 2021, Sivapalan et al., Clin Cancer Research, 2023). (**a**) These analyses showed that the concordance between radiographic (CR/PR vs SD/PD) and ctDNA responses depends on the fraction of patients with stable disease, a group that shows heterogeneity with respect to ctDNA responses across all cohorts analyzed (**b**). (**c**) Looking at differences in progression-free and overall survival within the radiographically stable patients across studies, ctDNA response accurately captured longer progression-free and overall survival (Anagnostou et al.: median OS = 13.6 for mR vs 13.7 for mPD, logrank p > 0.05; median PFS = 12.3 for mR vs 5.2 for mPD, logrank p = 9.8e-3. Murray et al.: median OS = 23.0 for mR vs 5.9 for mPD, logrank p = 8.0e-4; median PFS = 23.01 for mR vs 2.7 for mPD, logrank p = 4.8e-3). Stable disease annotation was not available for patients from Bratman et al. cohort, while only one patient who received immunotherapy had stable disease in the Sivapalan et al. cohort, resulting in exclusion of these cohorts from panels b and c. Median survival estimates were derived using survival fit function.

Patients with mR attained longer PFS than patients with mPD (median PFS 5.03 months versus 2.6 months for patients with ctDNA mR and mPD, respectively) (Fig. 5b). As radiographic response assessments are challenging in the context of immune checkpoint blockade and may not precisely reflect tumor burden dynamics^3^, we subsequently evaluated the association between ctDNA molecular responses and OS. Patients with mR had longer OS than patients with mPD (median survival not reached versus 7.23 months for patients with ctDNA mR and mPD, respectively) (Fig. 5c). In comparison, RECIST response less optimally distinguished patients with CR/PR from patients with SD with respect to PFS (8.31 months versus 4.27 months) and OS (not reached versus 16.89 months) (Extended Data Fig. 4). In post hoc analyses, we evaluated the heterogeneity of RECIST SD with respect to PFS and OS; patients with RECIST SD and mR (*n* = 2) had a PFS and OS of not reached versus 4.27 months and not reached versus 8.08 months, respectively, compared to patients with SD and mPD (*n* = 8). To put this in context and as part of the post hoc analyses, we evaluated ctDNA responses in patients with stable disease on immunotherapy from previously published studies^6,14,18,19^. These analyses further highlighted the heterogeneity of radiographic stable disease and the value of ctDNA molecular response to capture survival (Extended Data Fig. 5).

**Fig. 6:**
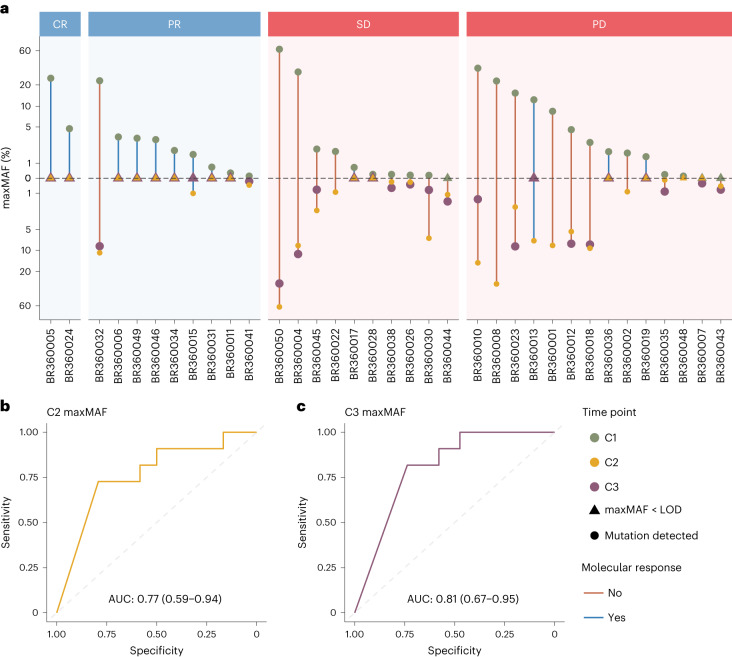
Depth of ctDNA response in association with RECIST radiographic response. **a**, All patients with complete radiographic response (CR) and six of nine patients with partial response (PR) showed ctDNA clearance (mR) during on-therapy timepoints (C2D1 and/or C3D1). In contrast, for patients with stable disease (SD) or progressive disease (PD), ctDNA clearance was much less frequent (two of 10 patients with SD and four of 14 patients with PD). The upper half of the vertical axis indicates the maxMAF observed in the baseline sample (green), whereas the lower half depicts the maxMAF in the on-therapy timepoints. maxMAF values are pseudo-log transformed for improved visual clarity. **b**,**c**, At each on-therapy timepoint, the fractional change in maxMAF compared to the baseline sample was used to predict the radiographic response at 12 weeks. Receiver operating characteristic (ROC) curves are shown, and point estimates for AUC along with 95% CIs are indicated. Change in maxMAF at C2D1 predicted RECIST response with an AUC of 0.77 (95% CI: 0.59–0.94), whereas change in maxMAF at C3D1 predicted RECIST response with an AUC of 0.81 (95% CI: 0.67–0.95). The dot sizes do not code for any data element displayed; rather, these are selected to visually maximize clarity, such that overlapping marks from distinct timepoints are not blocked and can be visually distinguished. LOD, limit of detection.

**Extended Data Fig. 6:**
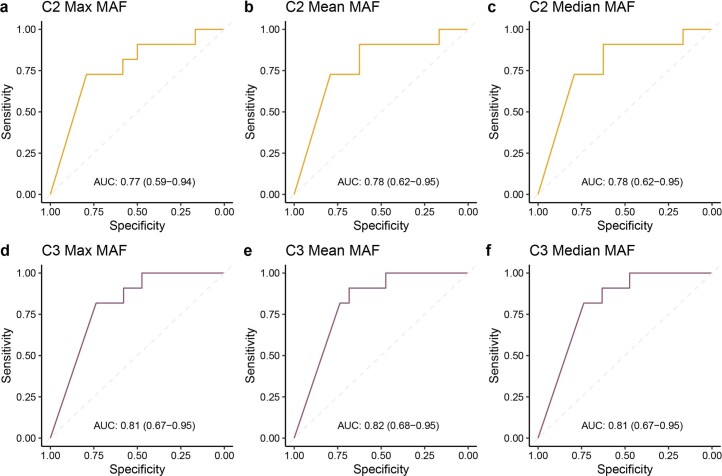
ROC curves for ctDNA change from C1 to C2 and C1 to C3 and RECIST response. MAF dynamics, computed from C1D1 to each on-therapy time point, were used to predict the radiographic response at 12 weeks by analyzing the maximum (**a, d**), mean (**b,e**), and median (**c, f**) MAF across all observed tumor-derived mutations. At each on-therapy time point, the change in MAF measure (maximum, mean, or median) compared to the baseline sample was calculated, and receiver operating characteristic (ROC) curves were drawn to assess performance. These analyses supported a slightly higher analytical performance of ctDNA molecular response at C3D1, with no significant differences noted when maximal, mean or median mutant allele frequencies of tumor-derived mutations were used to measure circulating tumor burden. Area under the curve along with 95% confidence intervals are reported.

We next explored the correlation of the degree of ctDNA reduction with radiographic RECIST response (Fig. 6a). The performance for the change in maxMAF, mean MAF and median MAF of tumor-derived variants compared to baseline (C1D1) was evaluated at C2D1 and C3D1. The depth of ctDNA response was predictive of radiographic RECIST response with an area under the receiver operating characteristic curve (AUC) of 0.77 (CI: 0.59–0.94) for C2D1 and 0.81 (CI: 0.67–0.95) for C3D1 ctDNA response assessments (Fig. 6b,c). Likewise, we found similar performance for the change in maxMAF, mean MAF and median MAF in predicting radiographic RECIST response at C2D1 (AUC: 0.78, CI: 0.62–0.95 and AUC: 0.78, CI: 0.62–0.95 for mean MAF and median MAF, respectively) or C3D1 (AUC: 0.82, CI: 0.68–0.95 and AUC: 0.81, CI: 0.67–0.95 for mean MAF and median MAF, respectively) (Extended Data Fig. 6).

**Extended Data Fig. 7:**
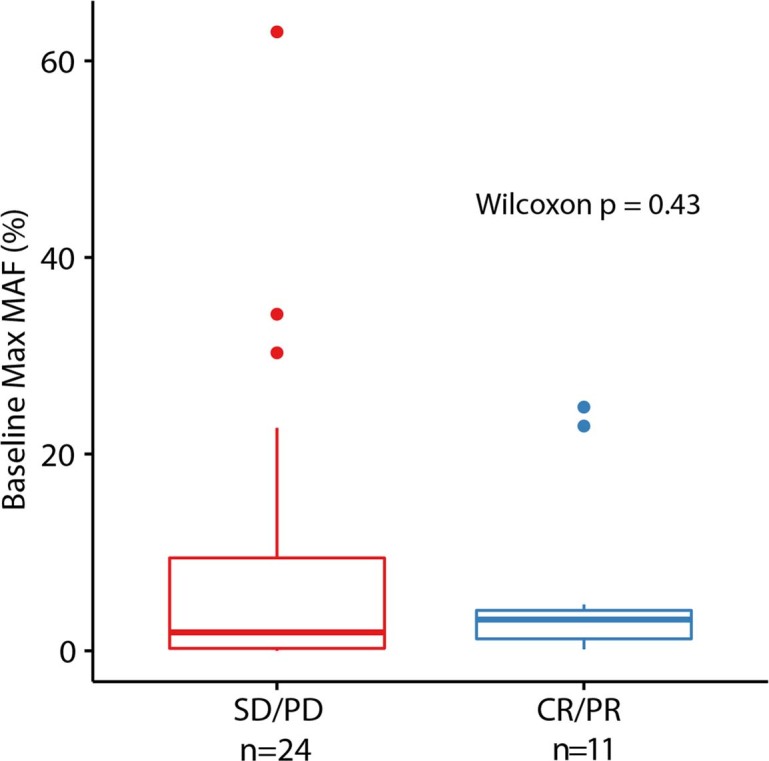
Baseline levels of circulating tumor burden differences based on radiographic RECIST response. There were no differences in MaxMAF values between patients with radiographic SD/PD (n = 24, non-responder group) compared to patients with radiographic CR/PR (n = 11, responder group) as assessed by the Wilcoxon test (two sided p = 0.43). Box plots depict the median value and hinges correspond to the first and third quartiles. The whiskers extend from the corresponding hinge to the furthest value within 1.5 * the interquartile range from the hinge.

**Extended Data Fig. 8:**
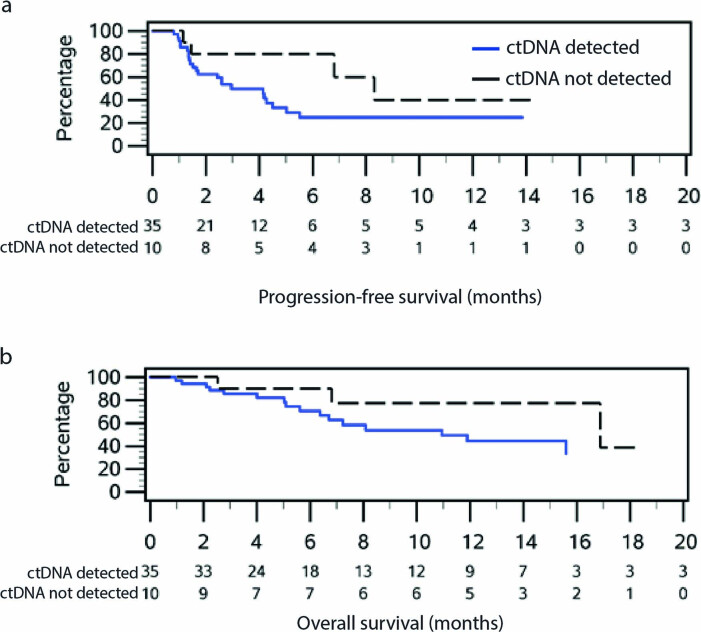
Survival outcomes by detectable vs. undetectable ctDNA status at baseline. (**a**) Patients with undetectable ctDNA at baseline (n = 10) had numerically longer PFS compared to patients with detectable ctDNA (n = 35, median PFS 8.31 vs 2.96 months). (**b**) Similar trends were noted for overall survival, such that patients with detectable ctDNA at baseline (n = 10) had numerically shorter OS compared to individuals with undetectable ctDNA at baseline (n = 35, median OS 10.94 vs. 16.89 months).

As part of post hoc analyses, we explored the association between baseline maxMAF and clinical outcomes. Although we did not identify any differences in baseline maxMAF between patients attaining a radiographic response (CR/PR) compared to patients in the SD/PD group (Wilcoxon *P* = 0.43, Extended Data Fig. 7), patients with undetectable ctDNA at baseline (*n* = 10) had numerically longer PFS and OS than patients with detectable ctDNA (*n* = 35, median survival 8.31 months versus 2.96 months for PFS and 16.89 months versus 10.94 months for OS, respectively; Extended Data Fig. 8). In evaluating the direct association of continuous baseline maxMAF with OS, we found that baseline maxMAF was not significantly associated with OS after adjustment for ctDNA molecular response status (hazard ratio (HR) = 0.986, 95% CI: 0.946–1.027, *P* = 0.56), indicating that ctDNA kinetics and molecular response was the most significant predictor of outcome (HR = 0.158, 95% CI: 0.052–0.48, *P* = 0.0063).

### Feasibility and concordance of tumor-agnostic WBC DNA-informed and tumor-informed approaches

As part of the study’s exploratory analyses, we evaluated whether a tumor-informed approach would more accurately capture ctDNA molecular responses. Of the 50 patients enrolled in the BR.36 study, only 19 (38%) had available matched tumor samples (Methods and Supplementary Tables 9 and 10). Furthermore, of the 19 patients with sufficient tumor samples sequenced, five had undetectable ctDNA, resulting in 14 (28%) patients with matched tumor NGS performed and detectable plasma variants. Of these, 11 (22%) patients had confirmed tumor-derived plasma variants by tumor NGS (Supplementary Table 10). We found a high concordance of 90.9% between the cellular origin of variants determined by the tumor-agnostic WBC DNA approach and confirmed tumor origin by matched tumor NGS. In the subset of patients with confirmed tumor origin of the plasma variants (*n* = 11), ctDNA molecular responses were 100% concordant between the tumor-informed and tumor-agnostic WBC DNA-informed approach. Notably, for patient BR360006, none of the plasma variants was detected in NGS of the matched tumor; however, by employing our tumor-agnostic approach, an *ATM* Q3000H mutation and a *KIT* C537W mutation were classified as tumor derived and evaluated for ctDNA kinetics. Similarly, for patient BR360026, there was no overlap between plasma variants and tumor NGS; nevertheless, the tumor-agnostic WBC-informed approach revealed a tumor-derived *KRAS* hotspot mutation that was tracked to determine ctDNA molecular response. A *KRAS* G12C was missed by tumor NGS for patient BR360036 but was detected, classified as tumor derived and tracked to determine molecular response. Although these exploratory analyses were limited by the lack of a trial-mandated tissue biopsy, our findings exemplify the challenges with the practical implementation of a tumor-informed liquid biopsy approach for assessing response to treatment as well as improved capture of tumor heterogeneity by liquid biopsies compared to archival tissue NGS analyses.

## Discussion

There is an unmet clinical need to implement real-time minimally invasive molecular analyses to capture therapeutic response and guide decision-making in the context of precision IO. We report here the findings of stage 1 of the Canadian Cancer Trials Group (CCTG) BR.36 trial, which aimed to define ctDNA molecular response, optimal timing of assessment and concordance with radiographic response. Employing a tumor-agnostic WBC DNA-informed panel NGS approach, ctDNA molecular response, defined as complete clearance of circulating tumor load after two cycles of pembrolizumab, was largely concordant with radiographic response assessments but, notably, was more informative in predicting OS. These findings are now incorporated into the second ctDNA molecular response-driven interventional randomized stage of the trial that will assess the value of adjusting therapy based on ctDNA response.

The landscape of IO is expanding rapidly; however, optimal patient selection for immunotherapy represents a critical challenge that is intensified by the heterogeneity of clinical responses and insufficiency of imaging to fully and timely capture changes in tumor burden and therapeutic response. Snapshot biomarkers currently used to guide therapy selection, such PD-L1 expression and TMB, rely on analysis of tumor tissue obtained through invasive single-region biopsies, which may not sufficiently capture the clonal complexity of tumors, may be problematic in the event of low tumor purity sampling and, importantly, do not capture the evolving tumor under the selective pressure of immunotherapy. These challenges highlight the urgent unmet need to develop molecularly informed strategies to improve patient selection, enable early and accurate response assessment and facilitate long-term response monitoring during immunotherapy. To this end, liquid biopsies are emerging as powerful minimally invasive approaches to monitor tumor burden as cancer cells go through immunotherapy-induced evolutionary bottlenecks and to rapidly and precisely capture therapeutic response.

We hypothesized that ctDNA molecular response can be used as an enrichment strategy to identify patients at high risk of clinical disease progression (those with molecular disease progression), thus limiting the heterogeneity of the target population, which, in turn, opens a therapeutic widow of opportunity for early intervention and interception of therapeutic primary resistance. An increasing number of studies support the role of ctDNA molecular response as an early endpoint of therapeutic response^6,7,8–14,20,21–23^. Despite the substantial progress made, there remain outstanding questions that need to be answered before implementation of ctDNA molecular responses in clinical decision-making, starting with what signifies a ctDNA molecular response. Although different measures have been used to capture circulating tumor burden during immune checkpoint blockade, including the mean^7,21^ or maximum^7,22,23^ MAF of tumor-derived alterations, a consensus definition of circulating tumor load is lacking. Similarly, several definitions have been proposed for ctDNA molecular response that are, in part, dependent on the NGS assay used and its sensitivity and negative predictive value^18^. Circulating tumor load regression more than 50%^10,12,24,25^, any ctDNA reduction^14^ or complete elimination^6,18^ have been proposed as potential definitions of ctDNA molecular response. In the ctMoniTR pooled analyses of ctDNA dynamics in IO-treated NSCLC, changes in maxMAF were shown to be the most predictive of therapeutic response^7^. The heterogeneity in cohort composition, treatment administered, timepoints analyzed and ctDNA methodology used represent critical challenges in these previous retrospective proof-of-concept studies as well as their pooled analyses. BR.36 was prospectively designed to address these challenges, and serial liquid biopsy analyses revealed that ctDNA complete elimination better captured clinical outcomes in the context of the NGS tumor-agnostic assay used in the study, which also incorporated patient-matched WBCs. Furthermore, we investigated whether mean, median or maximal MAF of tumor-derived variants can differentially serve as a proxy for circulating tumor burden, but we did not identify any differences, which is, in part, related to our definition of molecular response that entails ctDNA reduction to undetectable levels.

How early does molecular response occur? Several proof-of-concept studies have indicated that the optimal timepoint for ctDNA response lies between 4 weeks and 9 weeks from single-agent immune checkpoint blockade initiation^6,26^. In the first stage of BR.36, we found that the optimal timepoint is at C3D1, after two cycles of pembrolizumab; taking into account treatment delays, ctDNA response in BR.36 was determined at 8 weeks from treatment initiation. Importantly, understanding the true concordance between molecular and radiographic responses and how these differentially capture long-term outcomes in a clinical trial setting is imperative to support the clinical utility of ctDNA response. ctDNA molecular response may be most informative in characterizing the heterogenous group of patients with radiographically stable disease. ctDNA response has been shown to predict outcome with immune checkpoint blockade, such that patients with NSCLC with stable disease that cleared ctDNA had significantly longer PFS than patients who did not clear^6^. Patients with radiographic stable disease at first assessment who eventually attained a radiographic response have been reported to predominantly show ctDNA molecular responses^24^. Although a dedicated analysis of the heterogeneity of stable disease with respect to ctDNA response and long-term clinical outcomes was not included in the pre-specified analyses of the BR.36 study, patients within the stable disease subset had differential clinical outcomes that matched their ctDNA molecular response. Taken together, radiographic imaging may fail to timely detect the magnitude of therapeutic response for patients with stable disease, and ctDNA response may be of particular value in assessing therapeutic response in this setting.

Furthermore, we explored whether a tumor NGS-informed approach would be feasible and more informative than the tumor-agnostic WBC DNA-informed approach that we employed in BR.36. Tumor-informed liquid biopsy approaches for patients with metastatic disease may not be feasible from a tissue sufficiency standpoint and may restrict the evaluable plasma variants to these detected by single-region heterogenous tumor sample NGS. WBC DNA-informed liquid biopsy approaches can improve the specificity for tracking circulating tumor load compared to plasma-only approaches^27–29^ while retaining the advantage of capturing tumor heterogeneity and, as such, serve as a compelling alternative to tumor-informed approaches. WBC DNA-informed approaches also address the emergent challenge with biological noise in liquid biopsies, driven by mutations related to clonal hematopoiesis^28^. Nevertheless, it is plausible that alterations deemed as ‘tumor derived’ by a tumor-agnostic WBC DNA-informed approach may be derived from another lineage or represent ultra-low abundance clonal hematopoiesis alterations that were not detected by error-correction NGS of matched WBC. Notably, in the BR.36 cohort, only 22% of patients would have been evaluable by employing a tumor-informed liquid biopsy approach. Although the tissue–plasma NGS concordance analyses were limited by the lack of a trial-mandated tissue biopsy, it is important to note that, for the small fraction of patients with matched plasma and tumor NGS, we noted 100% concordance in ctDNA molecular responses, which further supports the validity of our tumor-agnostic WBC DNA-informed approach.

This study has several limitations, including the cohort size and the RECIST BOR of 32%, which likely reflected the real-world nature of the trial and reduced the statistical power to assess correlation of ctDNA with RECIST responses. Furthermore, as expected, approximately 20% of patients enrolled were not evaluable for ctDNA molecular response due to undetectable ctDNA, which represents a limitation given the shrinkage of the study population. Conceptually, a more sensitive multiplex polymerase chain reaction (PCR) tumor-informed liquid biopsy bespoke approach may reduce the number of cases with undetectable ctDNA; however, such an approach may not be feasible, especially in the context of a real-time ctDNA molecular response-adaptive interventional trial. Finally, the BR.36 study was powered to address the concordance between ctDNA and radiographic response, and, as such, assessment of additional endpoints may be limited by the study design. Per the trial design, we were limited in assessing the lead time between ctDNA response and RECIST response. ctDNA molecular response was assessed on C3D1 (2.1 months, accounting for delays within the BR.36 study cohort, otherwise on week 6 after pembrolizumab initiation), whereas best radiographic response was assessed at 12 weeks after treatment initiation. Although RECIST responses were evaluated at 6 weeks, together with ctDNA responses, BOR did not occur at 6 weeks, rather at 12 weeks; as such, ctDNA response provides the earliest and most accurate readout of best overall therapeutic responses.

Taken together, we show that ctDNA molecular response can identify patients with metastatic NSCLC less likely to attain favorable clinical outcomes with single-agent anti-PD-1 therapy, and this opens a therapeutic window of opportunity for treatment intensification for patients with molecular disease progression. Our findings were implemented in the design of the planned stage 2 of the BR.36 trial, which uses ctDNA detection after two cycles of standard-of-care pembrolizumab monotherapy to identify patients with metastatic NSCLC with PD-L1 ≥50% at high risk for disease progression, who are subsequently randomized to treatment intensification with pembrolizumab and chemotherapy versus continuation of pembrolizumab. Overall, our findings support the implementation of liquid biopsies in interventional IO clinical trials and further advance the evidentiary roadmap toward integration of ctDNA molecular responses in clinical decision-making for the increasing number of patients receiving immunotherapy.

## Methods

### Clinical trial design

The CCTG BR.36 trial (ClinicalTrials.gov ID: NCT04093167) was a phase 2, multi-center trial centrally activated on 17 October 2019. BR.36 stage 1 was designed as a single-arm, unblinded observational trial. BR.36 stage 2 is a randomized phase 2/3 trial that will evaluate whether adding chemotherapy to pembrolizumab for patients with advanced PD-L1^+^NSCLC who have persistent ctDNA at 6 weeks will result in better PFS and OS compared to patients who remain on pembrolizumab therapy until clinical progression. Primary endpoints are PFS and OS for the phase 2 and 3 portions, respectively (Extended Data Fig. 1). The trial was conducted according to principles of Good Clinical Practice and was reviewed and approved by ethics committees of six participating institutions, namely Johns Hopkins Hospital (Johns Hopkins Medicine Institutional Review Board), Ottawa Hospital Research Institute, Kingston Health Sciences Centre, Juravinski Cancer Centre, Princess Margaret Cancer Centre (Ontario Cancer Research Ethics Board) and BC Cancer Vancouver (University of British Columbia, British Columbia Cancer Agency Research Ethics Board). Written informed consent before trial participation was required for all patients. The first patient was enrolled on study on 26 May 2020, and the study was closed for accrual for stage 1 on 5 April 2022. An outline of the number of patients with samples available for analyses is shown in Fig. 1 and in the CONSORT diagram in Fig. 2. Two major protocol violations due to collection of the C1D1 blood sample after treatment initiation were reported. Pembrolizumab was administered as per local standard of care at 200 mg or 2 mg kg^−1^ intravenously (IV) every 3 weeks. After the first three cycles, investigators had the option of switching to pembrolizumab 400 mg or 4 mg kg^−1^ IV every 6 weeks. Patients continued on trial until radiographically defined progression or unacceptable toxicity or until maximum duration of treatment (24 months).

### Eligibility criteria

Full eligibility criteria were as follows:

Inclusion criteria

- Adult patients (≥18 years of age) with previously untreated, histologically or cytologically confirmed metastatic PD-L1^+^ (TPS ≥1% expression, PD-L1 test performed in a certified laboratory) NSCLC or stage III NSCLC if they are not candidates for surgical resection or definitive chemoradiation.
- ECOG PS of 0 or 1.
- Patients have to be eligible to receive treatment with pembrolizumab as standard of care, have an ECOG PS of 0 or 1 and have measurable disease and acceptable organ function.
- No prior systemic chemotherapy or immunotherapy for advanced metastatic NSCLC. Chemotherapy for non-metastatic disease (for example, adjuvant therapy) or immunotherapy for locally advanced stage III disease is allowed if at least 6 months have elapsed since the prior therapy and enrollment. Local therapy (for example, palliative extra-cranial radiation) is allowed as long as a period of 2 weeks has passed since completion. Patients must have recovered to ≤grade 1 from all reversible toxicity related to prior systemic or radiation therapy.
- Previous major surgery is permitted provided that surgery occurred at least 28 d before patient enrollment and that wound healing has occurred.
- Clinically and/or radiologically documented disease with at least one lesion measurable as defined by RECIST version 1.1.
- Imaging investigations, including computed tomography (CT) of the chest, abdomen and pelvis and magnetic resonance imaging (MRI) of the brain (if known brain metastases) or other scans as necessary to document all sites of disease must be done within 28 d before enrollment.
- Adequate hematology and organ function as defined below (must be done within 14 d before enrollment). Hematology: WBC ≥2.0 × 10^9^ per liter (2,000 per microliter), absolute neutrophils ≥1.5 × 10^9^ per liter (1,500 per microliter) and platelets ≥100 × 10^9^ per liter (100 × 10^3^ per microliter). Chemistry: bilirubin ≤1.5× the upper limit of normal (ULN)*, AST and/or ALT ≤3× ULN, <5× ULN for patients with liver metastases; serum creatinine or creatinine clearance** ≤1.5× ULN or ≥40 ml min^−1^ (* if confirmed Gilbert’s, eligible providing ≤3× ULN; ** creatinine clearance as calculated by Cockcroft and Gault equation below: females: GFR = 1.04 × (140 − age) × weight in kg serum creatinine in μmol L^−1^, males: GFR = 1.23 × (140 − age) × weight in kg serum creatinine in μmol L^−1^).
- Patients with large cell neuroendocrine carcinoma (LCNEC) and patients with clinically actionable *EGFR* or *ALK* genomic alterations, with symptomatic and uncontrolled brain metastases, who were pregnant/lactating or who were unwilling to use appropriate contraception were not eligible. Testing for *EGFR* and *ALK* is not required for patients with squamous histology.
- Patients have to consent to provision of a representative archival formalin-fixed, paraffin-embedded (FFPE) tumor block.
- Patients must consent to collection of liquid biopsy (blood) samples for ctDNA analysis by a CLIA central laboratory and for correlative analysis by a research central laboratory.
- Patient consent must be appropriately obtained in accordance with applicable local and regulatory requirements. Each patient must sign a consent form before enrollment to the trial to document their willingness to participate.
- Patients must be accessible for treatment and follow-up. Investigators must assure themselves that the patients enrolled on this trial will be available for complete documentation of the treatment, adverse events, collection of blood samples, response assessments and follow-up. Patients must agree to return to their primary care facility for response assessments as well as for any adverse events that may occur through the course of the trial.
- In accordance with CCTG policy, protocol treatment with pembrolizumab is to begin within two working days of patient enrollment.
- Women/men of childbearing potential must have agreed to use a highly effective contraceptive method. A woman is considered to be of ‘childbearing potential’ if she has had menses at any time in the preceding 12 consecutive months. In addition to routine contraceptive methods, ‘effective contraception’ also includes heterosexual celibacy and surgery intended to prevent pregnancy (or with a side effect of pregnancy prevention), defined as a hysterectomy, bilateral oophorectomy or bilateral tubal ligation or vasectomy/vasectomized partner. However, if at any point a previously celibate patient chooses to become heterosexually active during the time period for use of contraceptive measures outlined in the protocol, he/she is responsible for beginning contraceptive measures. Women of childbearing potential will have a pregnancy test to determine eligibility as part of the pre-study evaluation; this may include an ultrasound to rule out pregnancy if a false positive is suspected. For example, when beta-human chorionic gonadotropin is high and the partner is vasectomized, it may be associated with tumor production of hCG, as seen with some cancers. Patient will be considered eligible if an ultrasound is negative for pregnancy.

Of note, a PD-L1 TPS ≥50% was initially indicated as an inclusion criterion for the BR.36 trial, given the regulatory approvals in the United States and in Canada at the time of study design and activation. Throughout the duration of the BR.36 trial, the practice guidance changed in both the United Stated and Canada, allowing for pembrolizumab monotherapy for NSCLC with PD-L1 TPS ≥1%, and this was reflected in a trial amendment and revision of the eligibility criteria to include NSCLC with PD-L1 TPS ≥1%. This amendment was implemented in January 2021, after which 18 patients were enrolled. Despite the revised eligibility criteria, most patients enrolled in BR.36 had tumors with PD-L1 ≥50%, reflecting the preference for pembrolizumab monotherapy in this context given the higher magnitude of benefit.

Exclusion criteria

- Patients with a prior malignancy whose natural history or treatment has the potential to interfere with the safety or efficacy assessment of the investigational regimen are not eligible for this trial.
- Patients with symptomatic central nervous system (CNS) metastases and/or CNS metastases requiring immunosuppressive doses of systemic corticosteroids (>10 mg per day prednisone equivalents). Patients with known CNS metastases who are asymptomatic and on a stable dose of corticosteroids ≤10 mg per day prednisone equivalents before enrollment are eligible.
- Patients who are not suitable candidates for treatment with pembrolizumab according to the current guidance/indications described in the Product Monograph (Canada) or Drug Label (US), including, but not limited to, patients with active infection, autoimmune disease, conditions that require systemic immunosuppressive therapy (such as transplant patients) and patients with a history of severe immune-mediated adverse reactions or known hypersensitivity to pembrolizumab or its components. Patients with pre-existing conditions, such as colitis, hepatic impairment, respiratory or endocrine disorders (such as hypothyroidism or hyperthyroidism or diabetes mellitus), can be considered for enrollment to this study provided that pembrolizumab is administered with caution and patients are closely monitored.
- History of substantial neurologic or psychiatric disorder that would impair the ability to obtain consent or limit compliance with study requirements.
- Concurrent treatment with other anti-cancer therapy or other investigational anti-cancer agents.
- Pregnant or lactating women.

### Primary, secondary and exploratory endpoints

The primary objective of stage 1 of the BR.36 trial was to validate the concordance of ctDNA molecular response with radiographic RECIST version 1.1 response, ascertain its definition and identify the optimal timepoint for ctDNA molecular response. Secondary objectives included the evaluation of time to ctDNA molecular response, the correlation of ctDNA molecular response with PFS and OS and exploration of the degree of ctDNA reduction with clinical outcomes. The time to ctDNA molecular response was defined similarly based on changes in ctDNA levels, as described below. Tertiary objectives included the collection of archival tumor tissue samples and additional longitudinal plasma samples for future translational studies.

### Efficacy

Patients who received at least one cycle of pembrolizumab and had their disease re-evaluated after baseline were considered evaluable. Patients with objective disease progression before the end of cycle 1 were also considered evaluable. Using RECIST version 1.1 (ref. ^30^) and iRECIST^31^, radiographic restaging was initially performed every 6 weeks until week 54 and then every 12 weeks until disease progression. The protocol was amended on 14 October 2021 to allow imaging every 6 weeks until week 12 and then every 9 weeks until week 48 and then every 12 weeks until disease progression. RECIST BOR and iRECIST iBOR as well as first radiographic response (at 6 weeks from treatment initiation) were evaluated. The expected BOR rate was defined using the KEYNOTE-024 clinical trial as a reference^15^. Time to clinical response was defined from the date of starting the study treatment to the date of first documented response of CR/PR for those who achieved a CR/PR during the study, whereas it was censored at the date alternative therapy began for those who received non-protocol anti-cancer therapy before documented PD or the date of documented PD or date of death, whichever came first, or censored at the date of last disease assessment for those with SD and still alive at the end of the study.

### Safety

Safety was assessed using Common Terminology Criteria for Adverse Events (CTCAE) version 5. As patients were receiving pembrolizumab as standard of care, reporting of adverse events was required for all higher-grade toxicity (grades 3–5) and lower-grade toxicities if they led to treatment modifications (Supplementary Tables 1 and 2). All patients who received at least one dose of pembrolizumab were considered assessable for toxicity.

### Data collection and conventions for key data

Data were collected, entered and managed by the CCTG, according to the group standard data management procedures. A clinical data cutoff point was set once the 50th patient enrolled to the trial had been followed for 12 weeks and ctDNA molecular response and radiographic response had been determined. The clinical trial database was cleaned and locked on 20 September 2022. Baseline study evaluations were defined as those collected closest to and before or on the first day of study medication for study participants. The collection timepoints of samples analyzed for ctDNA molecular response were determined by collection date rather than the treatment cycle, as some cycles were delayed due to adverse events.

### Follow-up

The follow-up time was defined as the time from the date of registration to the date of last known alive status or at the date of death by the clinical data cutoff date.

### Sample collection

#### NGS of plasma-derived cell-free DNA and WBC-derived genomic DNA

Cell-free DNA (cfDNA) was isolated from serial plasma samples from 45 patients (*n* = 129) using the Circulating Nucleic Acid Kit (Qiagen), and the concentration was assessed using the Qubit dsDNA High-Sensitivity Assay (Thermo Fisher Scientific). Genomic DNA was isolated from patient-matched baseline WBC samples (termed WBC DNA, *n* = 45) using the QIAamp DNA Blood Mini Kit (Qiagen), and the concentration was assessed using the Qubit dsDNA High-Sensitivity Assay (Thermo Fisher Scientific). Subsequently, genomic DNA was sheared to a target size of approximately 200-bp fragments using Covaris focused ultrasonication. NGS libraries were prepared from cfDNA and fragmented genomic WBC DNA using a target of 40 ng of sample input through end-repair, A-tailing and adapter ligation with custom molecular barcoded adapters. Subsequently, libraries were PCR amplified, and target enrichment was performed through in-solution hybrid capture using the PGDx elio plasma resolve 33-gene panel^17,32^. Finally, libraries were pooled and sequenced with 150-bp paired-end reads using the Illumina NextSeq 550 platform. Although the liquid biopsy analyses in the first stage of the BR.36 trial were not performed in real time, the turnaround time of the assay is 6–8 d, which allows for the implementation of this approach in the second interventional stage of the trial. An overview of the plasma and WBC DNA samples sequenced, together with their sequencing quality control metrics, is shown in Supplementary Table 4. Somatic variant identification was performed using validated machine-learning-based algorithms, which have demonstrated high accuracy for somatic mutation detection and differentiating technical artifacts to enable analyses of single-nucleotide variants, small insertions/deletions, copy number amplifications, translocations and microsatellite instability^17,32–35^. The analytical performance of the PGDx elio plasma resolve assay was previously described in contrived samples, showing >99% specificity; >95% sensitivity for detection of alterations with a MAF of 0.25–1.0%; >95% repeatability and precision across different laboratory conditions; and >95% positive percent agreement and negative percent agreement compared to orthogonal methods across the majority of alteration types assessed^17^. To further expand these analyses beyond contrived samples and incorporate the ctDNA findings from the BR.36 study, we evaluated the variants detected and used for ctDNA molecular response assessment to calculate the estimated limit of detection based on the error-corrected coverage obtained through a binomial model. To determine sensitivity of the assay, we analyzed the set of 56 genomic positions harboring tumor-derived mutations at the baseline timepoint (C1D1). At each position, given the observed distinct coverage, the probability of observing a minimum of three error-corrected mutation counts at any given alteration MAF level was calculated using the binomial distribution. For each MAF level, the median of the estimated probabilities across the 56 positions was determined and used as an estimate for the assay sensitivity.

### NGS of tumor-derived genomic DNA

Thirty-four (68%) tumor samples were available, of which seven were macroscopically of insufficient quantity. FFPE tumor tissue sections for cases with available tumor material (*n* = 27) underwent hematoxylin and eosin staining and pathological review with a minimum of 20% tumor content required for sample testing and analysis. Two tumor samples had insufficient tumor purity upon pathology review, and tumor tissue macrodissection was performed to further enrich for tumor content for evaluable samples. Genomic DNA was extracted from FFPE tumor tissue using the Qiagen FFPE Tissue Kit and was sheared to a target size of approximately 200-bp fragments using Covaris focused ultrasonication. Five additional tumor samples were deemed insufficient due to suboptimal DNA yield. NGS libraries were prepared from fragmented genomic DNA using a target of 100 ng (minimum 50 ng) of sample input through end-repair, A-tailing and adapter ligation with custom barcoded adapters. Subsequently, these libraries were PCR amplified, and target enrichment was performed through in-solution hybrid capture using the PGDx elio tissue complete 505-gene panel^36^. Finally, libraries were pooled and sequenced with 150-bp paired-end reads using the Illumina NextSeq 550 platform. Average total and distinct coverage were 2,268× and 1,168×, respectively, and sequencing metrics are summarized in Supplementary Table 5. Somatic variant identification was performed using validated machine-learning-based algorithms, which have demonstrated high accuracy for somatic mutation detection and differentiating technical artifacts to enable analyses of single-nucleotide variants, small insertions/deletions, copy number amplifications, translocations, microsatellite instability and TMB^34,36–38^. Of the 20 sequenced tumor samples, 19 also had matched plasma NGS performed.

### Plasma variant cellular origin determination

A tumor-agnostic WBC DNA-informed approach was implemented to determine the cellular origin of variants detected by plasma NGS^18^ as follows. Variants were classified by origin in three categories: tumor derived, clonal hematopoiesis derived and germline. Non-cancer hotspot mutations with a MAF ≥25% in all plasma and WBC samples from the same individual were classified as germline and were removed from further analysis. Non-germline variants detected in plasma as well as in matched WBC DNA were classified as clonal hematopoiesis derived and were removed from further analysis, with the exception of cancer hotspot mutations. Cancer hotspot mutations (for instance, *KRAS* G12C) were considered tumor derived independent of detection in the matched WBC DNA samples, the latter likely indicating buffy coat contamination by circulating tumor cells or suboptimal pre-analytical processing. Supplementary Table 6 summarizes the cellular origin of variants detected by matched plasma–WBC DNA NGS. The frequency of alterations associated with clonal hematopoiesis was underestimated, as the targeted gene panel used in this study did not include *DNMT3A*, which is canonically mutated in clonally expanded hematopoietic cells. Although tumor NGS was not used to determine variant cellular origin or ctDNA molecular response, as described below, a limited exploratory tumor tissue–plasma concordance analysis was performed for 19 patients with matched tumor and plasma NGS.

### ctDNA molecular response evaluation

All patients who received at least one cycle of therapy and had ctDNA evaluated in at least one timepoint in addition to baseline were considered evaluable for ctDNA molecular response (*n* = 45). To alleviate technical challenges associated with the sensitivity of error-correction NGS at lower MAFs and according to previous studies^7,18^, the maxMAF of tumor-derived mutations was used as an indicator of cell-free circulating tumor load and computed for each timepoint analyzed. Similarly, given the broader CI in MAF estimates with decreasing MAF, around the assay limit of detection, a greater relative ctDNA reduction reflected in ctDNA clearance more accurately captures circulating tumor burden contraction^6,18,24^. Taken together, we defined ctDNA molecular response as maxMAF clearance; ctDNA molecular responses were assigned blindly with respect to radiographic responses and clinical outcomes. Having defined ctDNA molecular response as maxMAF clearance, we then determined the timepoint that this condition was met for individuals on the BR.36 trial, which was on C2D1 for 13 patients (maxMAF remained 0% at C3D1 for these individuals, with the exception of BR360048, for which C3D1 plasma collection was missed) and on C3D1 for two individuals, leading to the selection of C3D1 as the timepoint were the maxMAF clearance condition was met. Reduction of maxMAF to undetectable levels in C2D1 or C3D1 (ctDNA clearance at C2D1 and C3D1 or at C3D1) signified mR, whereas persistence of maxMAF at C3D1 indicated mPD^6^. The molecular response rate was calculated as the number of patients with mR divided by the number of all patients who were evaluable for ctDNA molecular response.

### Statistical analyses

The BR.36 study was powered to address the concordance between ctDNA and radiographic responses. The sample size was determined to ensure that the lower 95% confidence bound of the estimated sensitivity and specificity was higher than 50%, assuming the observed sensitivity and specificity were no less than 70%. The required sample size was 50 patients, assuming that 20% of patients will have undetectable ctDNA before therapy^6^, that the objective response rate to pembrolizumab is 45% (as reported in the KEYNOTE-024 trial)^15^ and that the sensitivity and specificity of the ctDNA molecular response are both no less than 70%; 18 responders would ensure that the lower bound of the 90% CI for estimated sensitivity is higher than 50%. Similarly, with 22 non-responders, the lower bound of the 90% CI for estimated specificity is higher than 50%. Among the patients with detectable ctDNA and evaluable ctDNA molecular response, the concordance of ctDNA molecular response with radiographic response and the sensitivity and specificity of ctDNA molecular response were estimated with 90% CI. Pre-specified analysis populations included the per-protocol population (that is, the eligible patients with detectable ctDNA and evaluable for ctDNA molecular response); all accrued patients in the trial; and the as-treated population (that is, all patients who received at least one dose of study treatment).

Discrete variables were summarized with the number and proportion of study participants falling into the category of interest. Continuous and ordinal categorical variables were summarized using the mean, median, standard error, minimum and maximum and interquartiles values where appropriate. For the time-to-event outcomes, the distributions were estimated with the Kaplan–Meier product limit method and summarized with median survival and 90% CI. Cox proportional hazards regression analysis was employed to assess the association of continuous maxMAF values with OS. The specificity and sensitivity and their CIs were estimated using the exact binomial distribution method.

We determined concordance between the depth of molecular response and radiographic RECIST response as follows. In each patient (*i*) and for each on-therapy timepoint (*C*^*x*^), the change in ctDNA level compared to baseline $({d}_{{C}^{x}}^{i})$ as

$${d}_{{C}^{x}}^{\,i}=\frac{\max \left(\left\{\,{f}_{{C}^{\,1}}^{\,i,\,j},\Big\vert,\,j\in \left\{1,\ldots ,n\right\}\right\}\right)-\max \left(\left\{\,{f}_{{C}^{\,x}}^{\,i,\,j},\Big\vert,\,j\in \left\{1,\ldots ,n\right\}\right\}\right)\,}{\max \left(\left\{\,{f}_{{C}^{\,1}}^{\,i,\,j},\Big\vert,\,j\in \left\{1,\ldots ,n\right\}\right\}\right)}$$

Here, ${f}_{{C}^{\,x}}^{\,j}$ indicates the MAF for mutation *j* of patient *i* in sample *C*^*x*^; negative values of ${d}_{{C}^{\,x}}^{\,i}$ indicate an increase in ctDNA level. In samples where the maxMAF of the baseline sample was 0, the ratio above is undefined. In such cases, the value of ${d}_{{C}^{\,x}}^{\,i}$ was set to the smallest value observed among the remaining samples from that timepoint in the cohort—that is, the largest increase compared to baseline. The continuous variable ${d}_{{C}^{\,x}}^{\,i}$ was used to predict a binary measure of RECIST radiographic response, where the responder group comprises patients with CR or PR and the non-responder group comprises patients with SD or PD. The AUC for the ROC curve was calculated to quantify performance (R version 3.6.1, pROC version 1.16.2).

### Reporting summary

Further information on research design is available in the Nature Portfolio Reporting Summary linked to this article.

## Online content

Any methods, additional references, Nature Portfolio reporting summaries, source data, extended data, supplementary information, acknowledgements, peer review information; details of author contributions and competing interests; and statements of data and code availability are available at 10.1038/s41591-023-02598-9.

### Supplementary information

Reporting Summary

Supplementary Tables 1–10 Supplementary Table 1. Toxicity/adverse events for the BR.36 study-evaluable patients (*n* = 50). Supplementary Table 2. Treatment-related adverse events of the BR.36 study-evaluable patients (*n* = 50). Supplementary Table 3. Overview of anatomic location of target and non-target lesions (all registered patients). Supplementary Table 4. Summary of RECIST and iRECIST responses in the trial cohort. Supplementary Table 5. Sequencing metrics for plasma and matched WBC DNA NGS. Supplementary Table 6. Plasma variants detected by NGS and their cellular origin. Supplementary Table 7. ctDNA kinetics and molecular response classification. Supplementary Table 8. Post hoc analyses of ctDNA response and RECIST response concordance using different baseline maxMAF thresholds. Supplementary Table 9. Sequencing metrics for tumor tissue NGS. Supplementary Table 10. Plasma variants detected in matched tumor tissue NGS.

## Data Availability

Next-generation sequencing data can be retrieved from the European Genome-phenome Archive (accession number EGAS00001007298). Clinical trial data can be requested through the Canadian Cancer Trials Group in accordance with its data sharing policy. Data access and contact details are described at https://www.ctg.queensu.ca/public/policies, with an expected turnaround time of 4–8 weeks.
