# Supplementary material for: ctDNA response after pembrolizumab in non-small cell lung cancer: phase 2 adaptive trial results
Source: Nat Med. 2023 Oct 9;29(10):2559–69. doi: 10.1038/s41591-023-02598-9 (PMC10579094; doi:10.1038/s41591-023-02598-9)
Supplement: Supplementary file 1 — Reporting Summary [file 41591_2023_2598_MOESM1_ESM.pdf]

nature portfolio

Corresponding author(s): Valsamo Anagnostou

Last updated by author(s): Sep 7, 2023

Reporting Summary

Nature Portfolio wishes to improve the reproducibility of the work that we publish. This form provides structure for consistency and transparency in reporting. For further information on Nature Portfolio policies, see our Editorial Policies and the Editorial Policy Checklist.

Statistics

For all statistical analyses, confirm that the following items are present in the figure legend, table legend, main text, or Methods section.

n/a	Confirmed	
☐	☒	The exact sample size (*n*) for each experimental group/condition, given as a discrete number and unit of measurement
☐	☒	A statement on whether measurements were taken from distinct samples or whether the same sample was measured repeatedly
☐	☒	The statistical test(s) used AND whether they are one- or two-sided<br>*Only common tests should be described solely by name; describe more complex techniques in the Methods section.*
☐	☒	A description of all covariates tested
☐	☒	A description of any assumptions or corrections, such as tests of normality and adjustment for multiple comparisons
☐	☒	A full description of the statistical parameters including central tendency (e.g. means) or other basic estimates (e.g. regression coefficient) AND variation (e.g. standard deviation) or associated estimates of uncertainty (e.g. confidence intervals)
☐	☒	For null hypothesis testing, the test statistic (e.g. *F*, *t*, *r*) with confidence intervals, effect sizes, degrees of freedom and *P* value noted<br>*Give P values as exact values whenever suitable.*
☒	☐	For Bayesian analysis, information on the choice of priors and Markov chain Monte Carlo settings
☒	☐	For hierarchical and complex designs, identification of the appropriate level for tests and full reporting of outcomes
☒	☐	Estimates of effect sizes (e.g. Cohen's *d*, Pearson's *r*), indicating how they were calculated

Our web collection on statistics for biologists contains articles on many of the points above.

Software and code

Policy information about availability of computer code

Data collection	Data were collected, entered and managed by the Canadian Cancer Trials Group (CCTG; Kingston, Ontario), according to the group standard data management procedures.
Data analysis	R version 3.6.1 was employed for statistical analyses.

For manuscripts utilizing custom algorithms or software that are central to the research but not yet described in published literature, software must be made available to editors and reviewers. We strongly encourage code deposition in a community repository (e.g. GitHub). See the Nature Portfolio guidelines for submitting code & software for further information.

Data

Policy information about availability of data

All manuscripts must include a data availability statement. This statement should provide the following information, where applicable:

- Accession codes, unique identifiers, or web links for publicly available datasets
- A description of any restrictions on data availability
- For clinical datasets or third party data, please ensure that the statement adheres to our policy

Next-generation sequence data can be retrieved from the European Genome-Phenome Archive (EGA; accession number EGAS00001007298). Clinical trial data can be requested through the Canadian Cancer Trials Group in accordance with its data sharing policy at https://www.ctg.queensu.ca/public/policies.

Human research participants

Policy information about studies involving human research participants and Sex and Gender in Research.

Reporting on sex and gender	Sex was determined by self-reporting, both females and males were enrolled in the study and sex was not a stratification criterion for stage 1 of the BR.36 trial.
Population characteristics	Population characteristics are summarized in Table 1 of our manuscript. Most patients were ever-smokers (98%), had stage IV NSCLC (98%) and no prior systemic therapy (92%); trial cohort consisted of 82% white, 52% female, 56% age 65 or older and 76% of participants had an ECOG performance status (PS) of 1 . Seventy six percent of tumors were adenocarcinomas and 96% had PD-L1 tumor proportion score of ≥ 50%.
Recruitment	50 patients were recruited based on the BR.36 clinical trial eligibility criteria as follows: Key eligibility criteria were adult patients with previously untreated, histologically or cytologically confirmed metastatic PD-L1 positive (TPS equal or greater than 1%) NSCLC or stage III NSCLC if they are not candidates for surgical resection or definitive chemoradiation. Patients had to be eligible to receive treatment with pembrolizumab as standard of care, ECOG Performance status 0 or 1 and with measurable disease and acceptable organ function. Patients with large cell neuroendocrine carcinoma (LCNEC) and patients with clinically actionable EGFR or ALK genomic alterations, symptomatic and uncontrolled brain metastases, pregnant/ lactating or unwilling to use appropriate contraception were not eligible. Furthermore, patients had to consent to provision of a representative archival formalin fixed paraffin tumor block. There was no appreciable bias in trial enrollment.
Ethics oversight	The trial was conducted according to principles of good clinical practices and reviewed and approved by ethics committees of six participating institutions, namely the Johns Hopkins Hospital (Johns Hopkins Medicine Institutional Review Board), Ottawa Hospital Research Institute, Kingston Health Sciences Centre, Juravinski Cancer Centre, Princess Margaret Cancer Centre (Ontario Cancer Research Ethics Board) and BC Cancer Vancouver (University of British Columbia - British Columbia Cancer Agency Research Ethics Board). Written informed consent before trial participation was required for all patients.

Note that full information on the approval of the study protocol must also be provided in the manuscript.

Field-specific reporting

Please select the one below that is the best fit for your research. If you are not sure, read the appropriate sections before making your selection.

☒ Life sciences ☐ Behavioural & social sciences ☐ Ecological, evolutionary & environmental sciences

For a reference copy of the document with all sections, see nature.com/documents/nr-reporting-summary-flat.pdf

Life sciences study design

All studies must disclose on these points even when the disclosure is negative.

Sample size	A total of 50 patients were accrued to the first stage of the BR.36 clinical trial. The sample size was determined to ensure that the lower 95% confidence bound of the estimated sensitivity and specificity were higher than 50% assuming the observed sensitivity and specificity were no less than 70%. The required sample size was 50 patients, assuming that 20% patients will have undetectable ctDNA pre-therapy6, the objective response rate to pembrolizumab is 45% (as reported in the KEYNOTE-024 trial)15 and that the sensitivity and specificity of the ctDNA molecular response are both no less than 70%; 18 responders would ensure that the lower bound of the 90% confidence interval (CI) for estimated sensitivity is higher than 50%. Similarly, with 22 non-responders, the lower bound of the 90% CI for estimated specificity is higher than 50%.
Data exclusions	Five patients were deemed not evaluable because of missed plasma collection or in-evaluable RECIST assessments. Of the 45 evaluable patients, 10 had undetectable ctDNA at all timepoints (no tumor-specific plasma variants detected), resulting in 35 patients with evaluable ctDNA and RECIST responses.
Replication	The liquid biopsy next-generation sequencing assay used in this clinical trial is a CLIA validated assay that is commercially available by Personal Genome Diagnostics, its analytical performance has been extensively validated with characteristics as follows: analytical sensitivity: 0.3-1.4% MAF, analytical specificity 99.998%.
Randomization	BR.36 stage 1 is an observational study and as such randomization was not applicable.
Blinding	Determination of ctDNA molecular responses was blinded to the radiographic responses.

Reporting for specific materials, systems and methods

We require information from authors about some types of materials, experimental systems and methods used in many studies. Here, indicate whether each material, system or method listed is relevant to your study. If you are not sure if a list item applies to your research, read the appropriate section before selecting a response.

Materials & experimental systems

n/a	Involved in the study
☒	☐ Antibodies
☒	☐ Eukaryotic cell lines
☒	☐ Palaeontology and archaeology
☒	☐ Animals and other organisms
☐	☒ Clinical data
☒	☐ Dual use research of concern

Methods

n/a	Involved in the study
☒	☐ ChIP-seq
☒	☐ Flow cytometry
☒	☐ MRI-based neuroimaging

Clinical data

Policy information about clinical studies

All manuscripts should comply with the ICMJE guidelines for publication of clinical research and a completed CONSORT checklist must be included with all submissions.

Clinical trial registration

ClinicalTrials.gov identifier NCT04093167

Study protocol

The full clinical trial protocol is provided as a separate attachment.

Data collection

BR.36 was centrally activated on October 17, 2019. The first patient was enrolled on study on May 26, 2020, and the study was closed for accrual for stage 1 on April 5, 2022. The clinical trial database was cleaned and locked on September 20, 2022. Data were collected, entered and managed by the Canadian Cancer Trials Group (CCTG; Kingston, Ontario), according to the group standard data management procedures.

Outcomes

The primary objective of stage 1 of the BR.36 trial was to identify the optimal timepoint for ctDNA molecular response and validate the concordance of ctDNA molecular response with radiographic RECIST 1.1 response. Among the patients with detectable ctDNA and evaluable ctDNA molecular response, the concordance of ctDNA molecular response with radiographic response, and the sensitivity and specificity of ctDNA molecular response was estimated with 90% CI. Pre-specified analysis populations included the per protocol population, i.e., the eligible patients with detectable ctDNA and evaluable for ctDNA molecular response, all accrued patients in the trial and the as-treated population (i.e. all patients who received at least one dose of study treatment). Secondary objectives included the evaluation of time to ctDNA molecular response, the correlation of ctDNA molecular response with progression-free and overall survival and exploration of the degree of ctDNA reduction with clinical outcomes. The time to ctDNA molecular response was defined similarly based on changes in ctDNA levels. Tertiary objectives included the collection of archival tumor tissue samples and additional longitudinal plasma samples for future translational studies.
